# Driving pressure: a marker of severity, a safety limit, or a goal for mechanical ventilation?

**DOI:** 10.1186/s13054-017-1779-x

**Published:** 2017-08-04

**Authors:** Guillermo Bugedo, Jaime Retamal, Alejandro Bruhn

**Affiliations:** Departamento de Medicina Intensiva, Pontificia Universidad Catolica de Chile, Marcoleta 367, Zip code 6510260 Santiago, Chile

Current guidelines for lung-protective ventilation in patients with acute respiratory distress syndrome (ARDS) suggest the use of low tidal volumes (Vt), set according to ideal body weight (IBW) of the patient [1], and higher levels of positive end-expiratory pressure (PEEP) to limit ventilator-induced lung injury (VILI) [2, 3]. However, recent studies have shown that ARDS patients who are ventilated according to these guidelines may still be exposed to forces that can induce or aggravate lung injury [4–6].

Airway driving pressure has received considerable attention after a publication by Amato et al. [7] of a complex and innovative statistical analysis of key randomized clinical trials that tested ventilatory settings in patients with ARDS. The analysis showed that driving pressure, as opposed to Vt and PEEP, was the variable that best correlated with survival in patients with ARDS [7]. Since this article, several authors have replicated this hypothesis in different clinical scenarios, to the point of suggesting that driving pressure may be a goal in itself [8].

In this Viewpoint, we review the physiological meaning of driving pressure, look at the current clinical evidence, and discuss the role of driving pressure when setting the ventilator, considering it more as a safety limit than an objective by itself. This discussion is restricted to patients undergoing controlled mechanical ventilation and without spontaneous breathing efforts. During spontaneous ventilation measurements of driving pressure will underestimate the real distending pressure of the respiratory system and it can, therefore, be misleading [9].

## Back to basics: what does driving pressure represent?

After the description of the baby lung concept [10], which revealed a physiologically small lungs in patients with ARDS, several studies in the 1990s tested the hypothesis that limiting Vt or airway pressures during mechanical ventilation might improve the outcome of these patients. In a pioneering single center study, Amato et al. were the first to show a reduction in mortality in this setting using a strategy based on maintaining low inspiratory driving pressures (lower than 20 cmH_2_O) along low Vt and high PEEP levels [11]. Shortly after, the large multicenter ARDSnet trial showed a decrease in mortality by nearly 25% in more than 800 patients with ARDS when using 6, instead of 12 mL/kg, IBW, confirming that Vt limitation is a fundamental strategy to improve survival of patients with ARDS [1].

However, some controversy was generated about the best way to titrate Vt: IBW, body surface area, lung size, airway pressures, etc. Going further back, the rationale of limiting Vt emerged from the description of the concept of baby lung, which tells us that in ARDS we are facing physiologically small lungs, and not rigid lungs as previously thought [10]. In Gattinoni et al.’s original study, while oxygenation and shunt were correlated with non-aerated tissue, static lung compliance was strongly correlated with the residual aerated lung volume [12], the volume of the baby lung.

With that being said, driving pressure (DP) is the difference between the airway pressure at the end of inspiration (plateau pressure, P_pl_) and PEEP [7, 13]. In turn, static compliance of the respiratory system (C_RS_) is the quotient between Vt and driving pressure. Ergo, by simple arithmetic, driving pressure is the quotient between the Vt and C_RS_ of the patient:

$$ \begin{array}{l}\mathrm{DP}={\mathrm{P}}_{\mathrm{pl}}-\mathrm{PEEP}\\ {}{\mathrm{C}}_{\mathrm{RS}}=\frac{\mathrm{Vt}}{{\mathrm{P}}_{\mathrm{pl}}-\mathrm{PEEP}}=\frac{\mathrm{Vt}}{\mathrm{DP}}\\ {}\mathrm{DP}=\frac{\mathrm{Vt}}{{\mathrm{C}}_{\mathrm{RS}}}\end{array} $$

Thus, driving pressure represents the Vt corrected for the patient’s C_RS_, and using driving pressure as a safety limit may be a better way to adjust Vt in order to decrease cyclic or dynamic strain during mechanical ventilation.

**Fig. 1:**
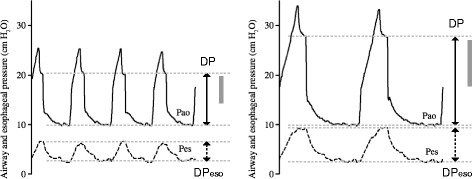
Airway (*P*_*ao*_) and esophageal (*P*_*eso*_) pressures in a patient with pneumonia and ARDS under volume-controlled ventilation with Vt 6 (left) and Vt 12 (right) mL/kg IBW and similar PEEP. Transpulmonary driving pressure (shown as *gray bars*) is the difference between airway driving pressure (*DP*, *solid arrows*) and esophageal driving pressure (*DP*_*eso*_, *dotted arrows*). Both airway DP and transpulmonary DP increased when using a higher Vt. Modified from [11]

Despite the fact that no study has prospectively tested the relationship between driving pressure and Vt, some scattered physiological data indicate it exists. In nine patients with ARDS, we applied both ventilatory strategies from the original ARDSnet study, 6 and 12 mL/kg IBW, at a constant PEEP (9 cm H_2_O), and minute ventilation. The use of lower Vt decreased airway driving pressure (11.6 ± 2.2 versus 22.7 ± 5.4, *p* < 0.01) and driving transpulmonary pressure (8.1 ± 2.2 versus 16.8 ± 6.0, *p* < 0.01) (Fig. 1), as well as cyclic recruitment-derecruitment and tidal hyperinflation [14]. Needless to say, Vt limitation decreased all the physical mechanisms involved in the genesis of VILI.

**Fig. 2:**
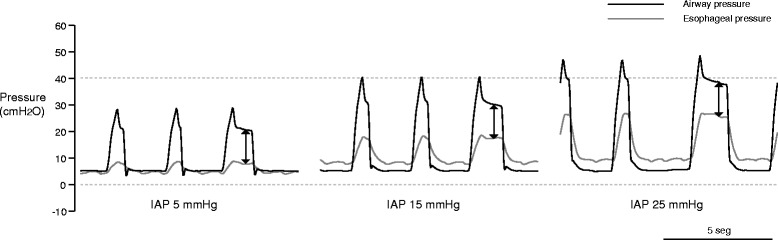
Airway (*black line*) and esophageal (*gray line*) pressure in an experimental model of abdominal hypertension secondary to pneumoperitoneum in pigs (data not published). During volume-controlled ventilation (Vt 10 mL/kg and PEEP 5 cm H_2_O), increases in intra abdominal pressure (IAP) from 5 (*left*) to 15 (*middle*) and 25 cm H_2_O (*right*) induced an increase in plateau pressure and driving pressure. However, driving transpulmonary pressure (*arrows*) remained constant

Transpulmonary driving pressure (the difference between airway plateau minus PEEP pressure and esophageal plateau minus end-expiratory esophageal pressure), when taking into account the chest wall elastance, could better reflect lung stress and be the safest way to titrate mechanical ventilation (Fig. 2) [13, 15, 16]. In this context, Chiumello et al. [13] conducted a retrospective analysis of 150 deeply sedated, paralyzed patients with ARDS enrolled in previous studies, in which a PEEP trial of 5 and 15 cm H_2_O was performed at constant Vt and respiratory rate. At both PEEP levels, the higher airway driving pressure group had a significantly higher lung stress, respiratory system, and lung elastance compared to the lower airway driving pressure group. More importantly, airway driving pressure was significantly related to lung stress (transpulmonary pressure), and driving pressure higher than 15 cm H_2_O and transpulmonary driving pressure higher than 11.7 cm H_2_O, both measured at PEEP 15 cm H_2_O, were associated with dangerous levels of stress.

Differences between transpulmonary driving pressure and airway driving pressure are mainly due to increases in chest wall elastance [15, 17]. Airway driving pressure may vary from minimal differences (skinny patient, pneumonia) to a large overestimation (morbid obesity, abdominal hypertension) of transpulmonary driving pressure. However, in the patient without spontaneous ventilatory activity, transpulmonary driving pressure will always be lower than airway driving pressure [13].

In summary, driving pressure during mechanical ventilation is directly related to stress forces in the lung. Sizing Vt in proportion to the size of the baby lung by targeting driving pressure, rather than to IBW, might better protect the lungs in patients with more severe lung injury and low end-expiratory lung volumes [8, 13].

## What is the current clinical evidence?

### Evidence relating driving pressure to outcomes

The association between driving pressure and outcomes was first described in 2002 [18]. In a prospective observational cohort of 235 patients with ARDS, Estenssoro et al. showed that driving pressure during the first week consistently discriminated between survivors and non-survivors, along with other variables, such as PaO2:FiO2 ratio and SOFA scores.

More than a decade later, the best evidence came from Amato et al. with the meta-analysis of nine prospective trials involving more than 3500 patients that showed that driving pressure was the physical variable that best correlated with survival in patients with ARDS [7]. More importantly, this association existed even though all the ventilator settings were lung-protective (plateau pressures ≤30 cm H_2_O and Vt ≤7 mL/kg IBW).

After the report by Amato, several authors confirmed the association of driving pressure with survival in patients with ARDS. In 56 ARDS patients from the EPVent trial [16], which tested the use of esophageal manometry in patients with ARDS, Baedorf Kassis et al. [19] found that utilizing PEEP titration to target positive transpulmonary pressures results in both improved elastance and driving pressures. The authors suggest that ventilation strategies leading to decreased driving pressure and elastance could be associated with improved survival.

In another secondary analysis of patients enrolled in two randomized controlled trials in ARDS patients, Acurasys [20] and Proseva [21], driving pressure was a risk factor for death, along with plateau pressure and C_RS_ [22]. More recently, in nearly 800 patients with moderate to severe ARDS managed with lung-protective ventilation, plateau pressure was slightly better than driving pressure in predicting hospital death [23]. The authors identified plateau and driving pressure cut-off values of 29 and 19 cm H2O, respectively, above which the risk of death increased.

Ultra-protective ventilation with extracorporeal lung support may help protect the lungs by decreasing Vt along driving pressure [24]. In a recent meta-analysis from nine studies, including more than 500 patients receiving extracorporeal membrane oxygenation (ECMO) for refractory hypoxemia, Serpa Neto et al. [25] showed that driving pressure during the first 3 days in ECMO had an independent association with in-hospital mortality. Although ECMO support allowed decreasing Vt to 4 mL/kg IBW and driving pressure in nearly 4 cm H_2_O, non-survivors still showed a higher driving pressure during ECMO (14.5 ± 6.2 versus 13.3 ± 4.8 cm H_2_O in survivors, *p* = 0.048).

In the largest observational study in nearly 2400 patients with ARDS, driving pressure of more than 14 cm H_2_O (and not Vt) was associated with an increased risk of hospital mortality in patients with moderate and severe ARDS [26]. The interesting data from this study indicates that there is still a significant potential for improvement by correcting modifiable factors associated with increased mortality, including driving pressure [27].

### Evidence relating driving pressure to pathophysiologic alterations

One of the problems when setting ventilation in ARDS patients is right ventricle (RV) overload, which relates to lung derecruitment and overdistension and has also been reported to be independently associated with a poor prognosis [28]. In a prospective observational study in 226 patients with moderate to severe ARDS ventilated with plateau pressures limited to 30 cmH_2_O and assessed with transesophageal echocardiography, cor pulmonale was detected in 49 patients (22%); higher driving pressures were an independent factor associated with cor pulmonale [29]. More recently, a driving pressure ≥18 cm H_2_O, a PaO2:FiO2 ratio <150 mmHg, and a PaCO2 ≥ 48 mmHg have been reported to promote RV failure in patients with ARDScaused by pneumonia [30].

There are also reports that describe the association of driving pressure with diaphragmatic function. In 107 patients on mechanical ventilation, Goligher et al. found an association between higher driving pressure and the decrease in thickness and contractile activity measured by ultrasound [8].

### Evidence relating modifications in driving pressure with outcome

Despite all the above evidence associating driving pressure with clinical and physiologic outcomes, no study to date has evaluated driving pressure as a primary goal during ventilatory setting in patients with ARDS. However, a few studies have analyzed the individual impact of specific interventions on driving pressure, and have related these changes to outcome.

In a recent prospective study in 200 patients with ARDS, Kacmarek et al. [3] showed that an open lung approach strategy (recruitment maneuver followed by a downward titration of PEEP), versus a more conservative PEEP strategy, improved oxygenation and decreased driving pressure, but without significant differences in survival.

In the surgical setting, a recent meta-analysis involving 17 clinical studies and 2250 patients showed that changes in the level of PEEP that resulted in an increase in driving pressure were associated with more postoperative pulmonary complications [31].

In the metanalysis of Amato et al. [7], when analyzing modifications to driving pressure which occurred as a result of specific changes in tidal volume or PEEP applied after randomization, those changes that led to a decrease in driving pressure were associated with a greater survival.

Although this evidence is rather weak to support a firm recommendation to target driving pressure as a primary goal in mechanically ventilated patients, we believe they constitute a promising basis for a future trial. In addition, they provide a clue for clinicians about how they might apply this new concept into clinical practice, while we await further evidence.

## Clinical use of driving pressure

Let’s compare theoretically two patients of similar age and phenotype with community acquired pneumonia and severe hypoxemia who are ventilated with the same level of Vt (6 mL/kg IBW) and PEEP (10 cm H_2_O). After an end-inspiratory occlusion maneuver, one patient has a plateau pressure of 22 cm H_2_O (driving pressure 12 cm H_2_O), while the other patient has 30 cm H_2_O (driving pressure 20 cm H_2_O). Clearly, the second patient has a lower C_RS_, and probably a worse prognosis. In this patient, after decreasing the Vt to 5 mL/kg and a PEEP titration to 14 cm H_2_O, plateau pressure drops down to 26 cm H_2_O. Will these two patients now, after achieving the same driving pressure of 12 cm H_2_O, have the same prognosis? Logic tends to suggest that this is not the case, as the patient with a higher severity of disease will require more adjunctive therapies, such as prone and neuromuscular blockade, but may still have a worse outcome.

As discussed, a high driving pressure is strongly associated with higher mortality. However, safe limits of driving pressure have not been identified and the suggested cutoffs vary from 14 to 18 cm H_2_O [26, 30]. In clinical studies comparing high versus low Vt ventilation in patients with ARDS, conventional non-protective strategies resulted in driving pressure greater than 20 cm HO, while protective ones were usually below 15–16 cm H_2_O. In contrast, in studies comparing high versus low PEEP, in which all groups limit Vt, mean driving pressures were well below 15 cm H_2_O (Table 1).

**Table 1:** Ventilatory parameters at 24 h and mortality in clinical studies comparing a protective strategy (Vt limitation) versus a control group (top panel), and a strategy of high PEEP versus low PEEP or minimal distension (lower panel) in patients with ARDS

Author	Year	N	Vt	P_pl_	PEEP	DP	Mort	Vt	P_pl_	PEEP	DP	Mort	Dif DP	*p* ^b^
Protective strategy								Control group						
Brochard	1998	108	7.1	25.7	10.7	15	46.6%	10.3	31.7	10.7	21	37.9%	6	NS
Stewart	1998	120	7.2	22.3	8.6	13.7	48.0%	10.8	26.8	7.2	19.6	46.0%	5.9	NS
Ranieri^a^	1999	44	7.6	24.6	14.8^a^	9.8	38.0%	11.1	31	6.5	24.5	58.0%	14.7	0.19
Brower	1999	52	7.3	27	9.3	17.7	50.0%	10.2	30	8.2	21.8	46.0%	4.1	NS
Amato^a^	1998	53	6	31.8	16.3^a^	15.5	38.0%	12	34.4	6.9	27.5	71.0%	12	<0.001
ARDSnet	2000	861	6.1	25	9.4	15.6	31.0%	11.9	33	8.6	24.4	39.8%	8.8	0.007
High PEEP								Low PEEP						
ALVEOLI	2004	549	6.1	27	14.7	12.3	27.5%	6.0	24	9.1	14.9	24.9%	2.6	NS
Mercat	2008	767	6.1	27.5	15.8	11.7	35.4%	6.1	21.1	8.4	12.7	39.0%	1.0	NS
Meade	2008	983	6.8	30.2	15.6	14.6	36.4%	6.8	24.9	10.1	14.8	40.4%	0.2	NS
Talmor^c^	2008	61	7.1	28	17	11	17%	6.8	25	10	15	39%	4.0	0.055
Kacmarek	2016	200	5.6	27.9	15.8	11.8	22%	6.2	25.2	11.6	13.8	27%	2.0	0.18

Driving pressure of the respiratory system (*DP*) is calculated as the difference between the plateau pressure (*P*_*pl*_) and PEEP. Note that a larger difference in DP between groups (Dif DP) is associated with differences in mortality

^a^ Ranieri [37] and Amato [11] studies also use high PEEP in the protective strategy

^b^ The *p* value refers to the differences in mortality (*Mort*) between groups

^c^ Ventilatory parameters at 72 h

**Fig. 3:**
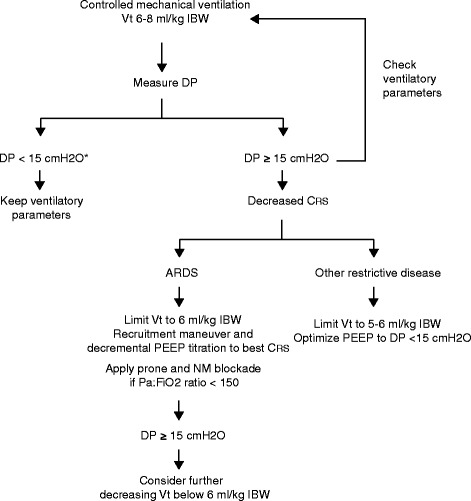
Suggested flowchart for adjusting ventilatory parameters according to driving pressure in patients requiring invasive mechanical ventilation. *The limit of 15 cm H_2_O is only speculative as no safe limit for driving pressure has been identified (see text). *Abbreviations*: *Vt* tidal volume, *IBW* ideal body weight, *DP* airway driving pressure, *C*_*RS*_ static compliance of the respiratory system, *NM* neuromuscular, *PaO2:FiO2 ratio* ratio of the partial pressure of arterial oxygen to the fraction of inspired oxygen

In the absence of prospective studies using driving pressure as a goal when setting the ventilator, we suggest that driving pressure should be used as a complement to, and not as a substitute for, Vt. Accordingly, we should maintain a Vt target of 6 to 8 mL/kg IBW, and then control its safety according to driving pressure (Fig. 3). Although there is insufficient evidence to suggest a specific cutoff value for driving pressure, we propose 15 cm H_2_O, not as a target, but as a safety limit. Probably most of the patients without ARDS will present a driving pressure below 10 cm H_2_O, reflecting a normal or near normal C_RS_ [31]. In contrast, in patients with moderate to severe ARDS or other restrictive diseases (pulmonary edema, large pleural effusions, interstitial disease, fibrosis, etc.), a driving pressure above 10 will be common, and it may reflect either a diminished C_RS_ or an inappropriate Vt/PEEP setting.

Driving pressure may be a valuable tool to set PEEP. Independent of the strategy used to titrate PEEP, changes in PEEP levels should consider the impact on driving pressure, besides other variables such as gas exchange and hemodynamics [3, 32, 33]. A decrease in driving pressure after increasing PEEP will necessarily reflect recruitment and a decrease in cyclic strain. On the contrary, an increase in driving pressure will suggest a non-recruitable lung, in which overdistension prevails over recruitment [34]. If after optimizing PEEP driving pressure remains above 15 cm H_2_O, we suggest further decreasing Vt below 6 mL/kg IBW (Fig. 3) [24]. In addition, an esophageal catheter may be considered to measure transpulmonary driving pressures.

## Conclusions

Airway driving pressure is the difference between plateau pressure and PEEP and represents the cyclic strain to which the lung parenchyma is subjected during each ventilatory cycle. It is a physiological way of adjusting Vt to the residual lung size (respiratory system compliance) of the patient, correlates directly with transpulmonary pressure, and is associated with survival in patients with ARDS [7]. Thus, setting ventilatory parameters to decrease driving pressure may have a role in improving outcomes in patients requiring mechanical ventilation.

However, driving pressure is only one of many variables involved in the mechanical power or energy applied to the lung parenchyma. Vt, flow, and respiratory rate have also been identified as causes of VILI [35, 36]. Further research will need to explore how all these factors behave in a particular patient.

In the meantime, we suggest adjusting ventilatory support with traditional protective parameters, Vt 6–8 mL/kg IBW and moderate PEEP levels, and adjusting them according to driving pressure, which should ideally be below 15 cm H_2_O, although this limit should be tested in future trials.

## Abbreviations

ARDS: Acute respiratory distress syndrome
C_RS_: Static compliance of the respiratory system
ECMO: Extracorporeal membrane oxygenation
FiO2: Fraction of inspired oxygen
IBW: Ideal body weight
PaCO2: Partial pressure of arterial carbon dioxide
PaO2: Partial pressure of arterial oxygen
PEEP: Positive end-expiratory pressure
P_pl_: Plateau pressure
RV: Right ventricle
VILI: Ventilator-induced lung injury
Vt: Tidal volume
